# Editorial: Transforming Youth Mental Health Treatment Through Digital Technology

**DOI:** 10.3389/fpsyt.2020.606433

**Published:** 2020-11-27

**Authors:** John F. M. Gleeson, Heleen Riper, Mario Alvarez-Jimenez

**Affiliations:** ^1^Healthy Brain and Mind Research Centre, School of Behavioural and Health Sciences, Australian Catholic University, Melbourne, VIC, Australia; ^2^Faculty of Behavioural and Movement Sciences, Vrije Universiteit (VU) University Amsterdam, Amsterdam, Netherlands; ^3^Orygen, Centre for Youth Mental Health, The University of Melbourne, Parkville, VIC, Australia

**Keywords:** mental health services, youth mental health, digital technology, eHealth, early intervention

The historical perspective provides a powerful vantage point for recognizing progress, unfulfilled promises, potential pitfalls, and current opportunities. Leonard Bickman recently provided an example which is highly salient to the contents of this Research Topic in his conceptual paper entitled “Improving Mental health Services: A 50-year journey from randomized experiments to artificial intelligence and precision mental health” (1). Many of his observations align with our view that without significant reform current mental health service models, designed in their essential form between 50 and 75 years ago, are no longer fit for purpose given the poor access to care and the problems with their effectiveness (1).

In many jurisdictions around the world community mental health care can be characterized as a moribund system staffed by compassionate, dedicated, and competent clinicians, a status which is increasingly acknowledged even by the governments that fund them. For example, the state of mental health services has led to the first Royal Commission into mental health services in Australia (2). In launching the Royal Commission the local state political leader, Premier Daniel Andrews, declared that the Victorian mental health system is “a broken system and until we acknowledge that and set a course to find those answers and a practical plan for the future, people will continue to die, people will continue to be forever diminished.” (2) p. 11.

With the wisdom of hindsight, the era of deinstitutionalization and the community mental health service reform was founded upon incorrect assumptions about the resources required, especially for those with complex comorbidities (3). Without further reform the gap between demand for mental health treatment and its supply will be intensified by the competing pressures on public funds as the world enters a fragile phase of economic and mental recovery from the COVID-19 pandemic. COVID-19 has also cruelly revealed that the expectation of inevitable business and institutional continuity is a false and dangerous implicit assumption. We have been abruptly reminded of the critical importance of sustainable platforms for service delivery and the critical importance of ready access during crises to redundant capacity, especially in health care. It's clear that in a post-COVID-19 world that current models of mental health service delivery cannot be adequately scaled to reliably meet the needs of young people and their families in the twenty-first century and beyond. Therefore, modified and alternative models of providing mental health assessment and intervention for young people are critically needed. Bickman provided a glimpse of the array of possible solutions that technology could lend to the next wave of reform (1). The papers in this Research Topic illustrate many of those possibilities and highlight the creativity and breadth of research into digital transformation of youth mental health and well-being.

The current problem of the unsustainable demand on mental health services can be reduced via effective preventive technologies which requires clinicians to pivot toward neglected environments and populations at heightened risk. There are three examples in this Research Topic including a focus on schools (O'Dea et al.), University students (Fleming et al.), and some thoughtful commentary on the prospects for preventive intervention in bipolar disorder (Murray).

As Bickman highlighted, significant work is required to harness the affordances of technology in clinical assessment, which offers the possibility of increased scale, intensity, and ecological validity. Van Dam et al.'s demonstration of the feasibility of using emojis is a creative example of a simple and familiar tool for young people for real-time assessments.

As David Mohr and colleagues have cogently highlighted, the hard won gains made in establishing feasibility and effectiveness of novel digital mental health interventions are often poorly translated into real-world outcomes (4). In line with Mohr's argument that co-design with clinical services is critical for future success, Bucci et al. highlight the importance of investing in the understanding of the frontline clinical workforce's perceptions and needs in relation to implementation of digital tools in early interventions services. Peck et al. also illustrate this approach including the involvement of peer support staff in co-design—a highly promising avenue for improving scalability of models of youth mental health service delivery.

Discerning the most appropriate treatment targets for digital interventions is a critical challenge for the field. We note recent calls for a shift away from group-based symptom reduction to trans-syndromal phenomena and psychological well-being (5), which we believe can be facilitated by the deployment of digital technology and partnering with young consumers and their carers in the collection of intensive ecologically valid data across time. Consistent with this call, in this special edition Kim and colleagues report preliminary effectiveness data from a controlled study of eye-gaze feedback to address the important transdiagnostic target of attention training (Kim et al.). Lim et al., also reports some highly promising data in relation to the critical transdiagnostic target of loneliness in youth.

Bickman foreshadowed an increasing roll for precision mental health by leveraging big data (1). The potential for individualized interventions stemming from assessment and data capture can be seen in Iorfino et al.'s description of their new platform for implementation in youth services. A significant challenge for this cutting-edge field will be determining the criteria for achieving precision in meeting the needs of the individual young person. Efficiency will also be an important consideration in further reform of youth mental health services which raises tension between allowing a 1,000 digital flowers to bloom, vs. adapting for mental health care existing digital resources. Thompson et al. present an intriguing example, embedding a mental health intervention within a familiar digital environment.

The next 5 years of research progress in digital interventions for youth mental health will be intriguing. Whilst reluctant to make predictions, we would like to proffer critical priorities. First, we argue for the need to enhance the science of digital engagement to establish the most effective strategies to optimize involvement of young people in digital mental health care and to promote their re-engagement during periods of setback. COVID-19 has also brought the preventive role of passive forms of engagement via passive sensing into public awareness and in youth mental health care the challenge will be to optimize both its effectiveness and the autonomy of the end user to create empowerment and autonomy. Another critical priority will be determining what are the most viable targets for behavior change via digital interventions and which behavior change techniques are most readily utilized via technology for young people to address those targets? Can we scale up effective behavior change strategies using natural language processing and machine learning? Can we realize the goal of precision interventions by using AI to match individuals via real-time assessment to personally salient transdiagnostic treatments? Perhaps the most urgent question is how can we optimize the integration of in-person and digital interventions as well as blended formats combining the best of both worlds (face-to-face and digital components) in integrated treatment protocols, e.g., by providing clinicians with real-time feedback on the fidelity of their interventions, by reinforcing therapy homework, and using real-time assessment to recognize when intensive in-person intervention is needed? Co-design will be critical to the success of these endeavors.

With the inevitable false starts, setbacks and success ahead it will be critical to retain the past lessons from deinstitionalization by taking account of the full complexity of digital implementation whilst also holding public and private institutions to account in building out a viable model. Finally, as researchers and clinicians continue to explore the boundaries of what's possible it will remain important to hold onto another important reminder from the COVID-19 pandemic—that technology can only reach so far in meeting the universal and fundamental human need for connection that will always require a compassionate, intelligent, and competent physical human presence.

## Author Contributions

JG drafted the editorial. HR and MA-J provided editorial input. All authors contributed to the article and approved the submitted version.

## Conflict of Interest

The authors declare that the research was conducted in the absence of any commercial or financial relationships that could be construed as a potential conflict of interest.
